# Preparation, Surface and Pore Structure of High Surface Area Activated Carbon Fibers from Bamboo by Steam Activation

**DOI:** 10.3390/ma7064431

**Published:** 2014-06-12

**Authors:** Xiaojun Ma, Hongmei Yang, Lili Yu, Yin Chen, Ying Li

**Affiliations:** Department of Wood Science and Technology, Tianjin University of Science & Technology, Tianjin 300222, China; E-Mails: furniture2004@163.com (H.Y.); yulilucky@tust.edu.cn (L.Y.); chenyin880828@163.com (Y.C.); liyingha163@163.com (Y.L.)

**Keywords:** activated carbon fibers, bamboo, pore size distribution, adsorption, X-ray diffraction

## Abstract

High surface area activated carbon fibers (ACF) have been prepared from bamboo by steam activation after liquefaction and curing. The influences of activation temperature on the microstructure, surface area and porosity were investigated. The results showed that ACF from bamboo at 850 °C have the maximum iodine and methylene blue adsorption values. Aside from the graphitic carbon, phenolic and carbonyl groups were the predominant functions on the surface of activated carbon fiber from bamboo. The prepared ACF from bamboo were found to be mainly type I of isotherm, but the mesoporosity presented an increasing trend after 700 °C. The surface area and micropore volume of samples, which were determined by application of the Brunauer-Emmett-Teller (BET) and t-plot methods, were as high as 2024 m^2^/g and 0.569 cm^3^/g, respectively. It was also found that the higher activation temperature produced the more ordered microcrystalline structure of ACF from bamboo.

## 1. Introduction

Activated carbon fibers (ACFs) are relatively novel fibrous adsorbents that show important advantages with respect to conventional activated carbons due to the easily handled and the faster adsorption kinetics. Thus, it has a high adsorption capacity for pollutants such as methylene blue, phenolic compound, lead ions and vapors of some volatile organic compounds [1]. Most ACFs have been synthesized from precursors based on the fossil fuels. The shortage of these resources necessitates an improvement in ACFs production which highly depends on chemicals [2,3,4]. Therefore, it is necessary to find cheap and renewable new types of precursors. In recent years, growing research interest has been focused on various natural materials, which can be used for the production of ACFs with high adsorption capacity. Some of the natural materials include Kenaf [5], cotton stalk [6], coconut shell [7], wood [8,9] and paper [10]. However, limited reports were found using bamboo as raw materials for the preparation of high surface ACFs, except activated carbon [11,12,13,14]. 

Biomass is an abundant and renewable carbon source with many ecological advantages. Moreover, bamboo is readily available and a fast growing resource which offers great potential application as an alternative to wood. Bamboo is mainly composed of cellulose, lignin and hemicellulose. However, cellulose fibers from bamboo are too short for the texture production [15], which is the main purpose of ACFs. As a result, it is important to study the potential of bamboo fiber as ACFs precursor. Liquefaction technique can completely convert wood or bamboo into useful liquid chemical raw [16,17,18], which greatly improves the utilization of these materials, and provides a new method for the preparation of bamboo based ACFs. Moreover, some studies have reported the preparation of carbon fibers from liquefied wood instead of fossil fuels [19,20].

In the present study, ACF prepared from bamboo (BACFs) were obtained by steam physical activation after liquefaction and curing. The influences of activation temperature on the pore structure (specific surface area, pore volume, and pore size distribution) were examined. In addition, the microstructure and the surface chemistry of the BACFs were investigated. The adsorption performance of BACFs, the iodine and methylene blue (MB) adsorption of BACFs under different activation temperatures were also studied.

## 2. Results and Discussion

### 2.1. Morphological Characteristics of BACFs

**Figure 1:**
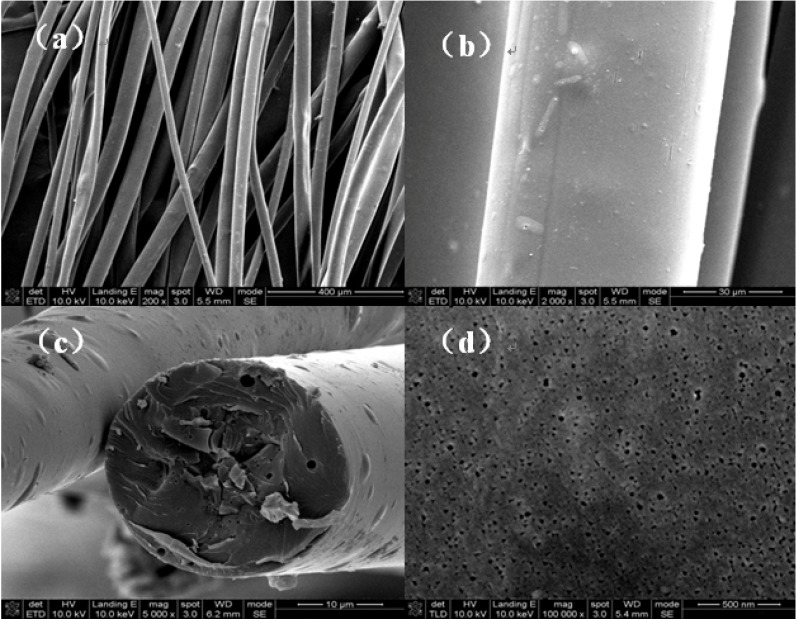
SEM micrographs of activated carbon fibers prepared from bamboo (BACFs): (**a**) and (**b**) side surface; (**c**) cross section; (**d**) side surface.

SEM micrographs of BACFs are shown in Figure 1. From these figures it is clear that the rough surfaces of BACFs can be easily detected as knots and bubbles (see Figure 1a,b). Because of the different crosslinkage degree during curing treatment, there are a loose core and a denser edge of fiber cross-section, resulting in the appearance of skin-core structure. Moreover, many pores are observed in the cross section (see Figure 1c), due to gas caused in the process of synthesis of spinning solution. In addition, more developed pore structure can be observed on the surface of BACFs at 850 °C (see Figure 1d).

### 2.2. The Iodine, MB Adsorption and Yield Rate of BACFs

**Figure 2:**
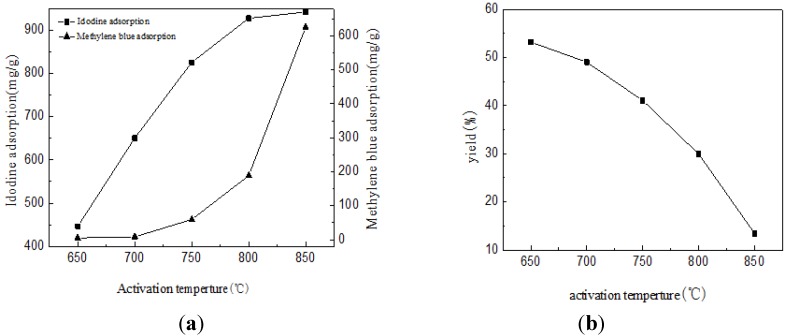
The iodine, methylene blue (MB) (**a**) adsorption and (**b**) yield of BACFs.

Figure 2 shows the iodine, MB adsorption and yield rate curve of BACFs under different activation temperatures. As can be seen, BACFs showed higher adsorption capacities, which increased with the rise of the activation temperature. The iodine adsorption values of BACFs increased more significantly than MB adsorption values in the activation temperature range of 650–800 °C, while the opposite trend can be observed over 800 °C. This implies that the pore size distributions of BACFs have significantly changed with the increasing of activation temperature, resulting on the more amounts of mesopores and macropores. Enhanced adsorption of methylene blue that benefited from the addition of mesopores has also been reported [21,22]. The iodine and MB adsorption values of BACFs at 850 °C are 942 mg/g and 626 mg/g, respectively. As seen in Figure 2, with increased activation temperature, the yield rate of BACFs gradually decreases. The yield rate of BACFs at 850 °C is 13.42%. It is also clear that the porous structure of BACFs could be obtained by improving the activation temperature, but the cost is high due to the low yield.

### 2.3. XRD Analysis of BACFs

**Figure 3:**
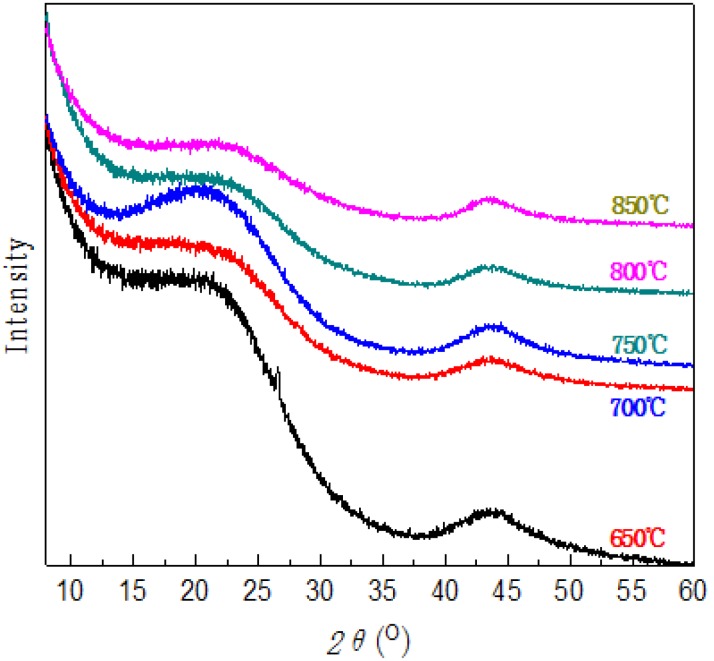
XRD of BACFs at various temperatures.

Figure 3 shows XRD patterns of BACFs at various activation temperatures. With increased activation temperature, the 002 peak begins to shift towards higher angle and becomes broader, corresponding to smaller sized crystallites. Above 650 °C, the samples presents (100) diffraction peak (2θ = 43°). Finally, X-ray diffractograms for BACFs show a similar diffraction pattern. The XRD structure parameters of BACFs under various activation temperatures are shown in Table 1. It can be observed with increased activation temperature, that the value of *d*_002_ gradually decreases, whereas the values of the crystallite sizes *L*_a_, *L*_c_ and *L*_c_*/d*_002_ value slightly increase. At the same time, the *g* value corresponding to the change degree of graphitization structure increases. As can be seen, the *L*_c_ value is increased from 0.88 to 2.39 nm, while the *L*_c_*/d*_002_ value is increased from 2.17 to 2.98 nm; indicating that a higher activation temperature produces thicker crystal and a denser structure [23,24].

**Table 1:** Structure parameters of XRD for BACFs.

Temperature (°C)	*d*_002_/nm	*L*_c_/nm	*L*_a_/nm	*L*_c_/*d*_002_	*g*/%
650	0.4072	0.88	1.83	2.17	−7.25
700	0.4053	1.98	1.98	2.37	−7.14
750	0.4053	2.06	2.06	2.46	−7.14
800	0.3930	2.11	2.11	2.60	−5.69
850	0.3879	2.39	2.39	2.98	−5.10

### 2.4. XPS Analysis of BACFs

Elemental analysis was carried out to obtain the compositions of C, O and P atoms in the fibers. As seen from Table 2, element C was the most abundant constituent in BACFs. With increased activation temperature, there was an increasing in C content, probably due to the release of volatiles. The presence of P could also be explained from the addition of phosphoric acid catalyst for liquefaction. Activation treatment has provoked a progressive release of oxygen, with a remarkable loss at 850 °C. This implies that the fraction of oxygen was located around the pore entry, hence the extension of micropores at 850 °C resulted in a large decrease in oxygen, corresponding to a much higher C/O atomic ratio [25,26].

**Table 2:** Elemental composition of the surface of BACFs.

Temperature (°C)	C (wt%)	O (wt%)	P (wt%)	C/O
650	80.72	18.91	0.37	4.26
700	82.85	16.71	0.40	4.95
750	83.08	16.83	0.08	4.94
800	84.40	15.30	0.30	5.51
850	88.55	11.19	0.26	7.91

**Figure 4:**
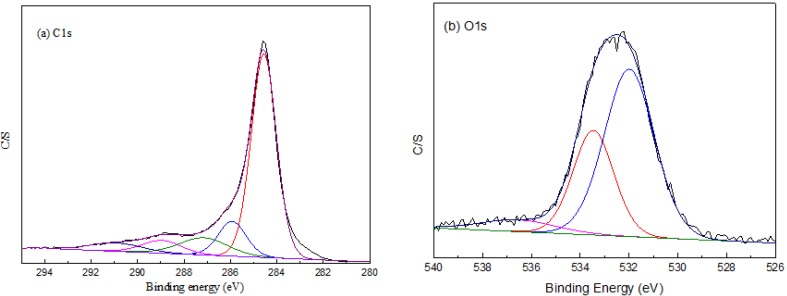
High-resolution X-ray photoelectron spectroscopy (XPS)fitted (**a**) C 1s and (**b**) O1s spectra of BACFs.

To obtain information about the chemical composition of the fiber surface and the binding characteristics of the elements at the surface, the measurements of the high-resolution XPS C 1s spectra were carried out. The bands obtained by spectral deconvolution of the C1s peak are shown in Figure 4, and the functionalities attributed to each are presented in Table 3. The same functionalities were detected for both types of fibers, namely hydroxyl or ether groups, quinone type groups and carboxylic acid. The C1s spectra have each been resolved into five individual component peaks that represent graphitic carbon (CP1, 284.6 eV), and carbon present in phenol, alcohol, ether or C=N groups (CP2, 285.8–286.0 eV), carbonyl or quinine groups (CP3, 286.7–287.5 eV), carboxyl, lactone, or ester groups (CP4, 288.3–289.0 eV), and carbonate groups (CP5, 289.7–290.8 eV) [27,28,29].

**Table 3:** XPS results of the peak fits of the C1s and O1s regions.

Temperature (°C)	Graphite (C_P1_)		C–OH (C_P2_)		C=O (C_P3_)		C–OOH (C_P4_)		CO_3_^2−^, CO, CO_2_ (C_P5_)	
	*BE* (eV)	*M* (%)	*BE* (eV)	*M* (%)	*BE* (eV)	*M* (%)	*BE* (eV)	*M* (%)	*BE* (eV)	*M* (%)
650	284.6	68.5	286.0	14.7	287.2	7.0	288.7	6.2	290.1	3.7
700	284.6	70.9	286.0	9.6	286.8	6.8	288.5	8.7	290.2	4.5
750	284.6	69.4	285.8	10.8	286.7	6.5	288.3	7.2	289.9	6.1
800	284.6	66.0	286.0	15.5	287.5	7.5	288.9	3.2	289.7	7.8
850	284.6	65.3	286.0	12.4	287.2	10.5	289.0	6.4	290.8	5.4

It can be seen from Table 3 that there was a decrease in the relative content of graphitic carbon and an increase in the relative content of carbon bonded to oxygen-containing functions after heat treatment. Aside from the graphitic carbon, C–OH, C=O and C–OOH groups were the predominant functions on the surface of ACFs. The relative composition of the fibers’ surface was similar to the published work [30]. The surface oxides on BACFs change from C–OH or C=O to C–OOH before heating up to 700 °C. However, the oxygen-containing groups change from C–OH to C=O or C–OOH after heating up to 800 °C. A slight increase of surface oxides on BACFs after heat treatment may cause by the formation of oxygenic functional groups, which the activating agent reacts with the reactive centers such as disorganized carbons, carbons with heteroatom and carbons on graphene edges, creating new pores and widening the existing ones during the activation [31].

### 2.5. N_2_ Adsorption of BACFs

**Figure 5:**
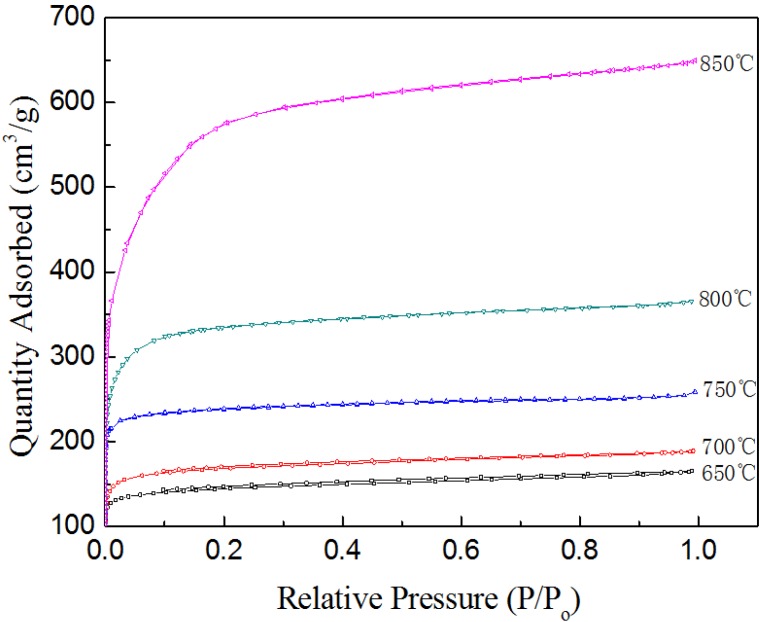
N_2_ adsorption isotherms at −196 °C of BACFs at various temperatures.

The N_2_ adsorption-desorption isotherms for BACFs obtained at various final activation temperatures were investigated. Figure 5 shows the adsorption-desorption isotherms of BACFs prepared at various temperatures. As can be seen from Figure 5, at low relative pressures, a rapid increase in the adsorption–desorption isotherms is observed, which is followed by a plateau at higher relative pressures, indicating a type I isotherm according to the IUPAC classification [32,33]. The type I isotherm represents a material with microporous structure. The major uptake occurs at low relative pressures indicating the formation of highly porous materials with narrow pore size distribution [34].

**Figure 6:**
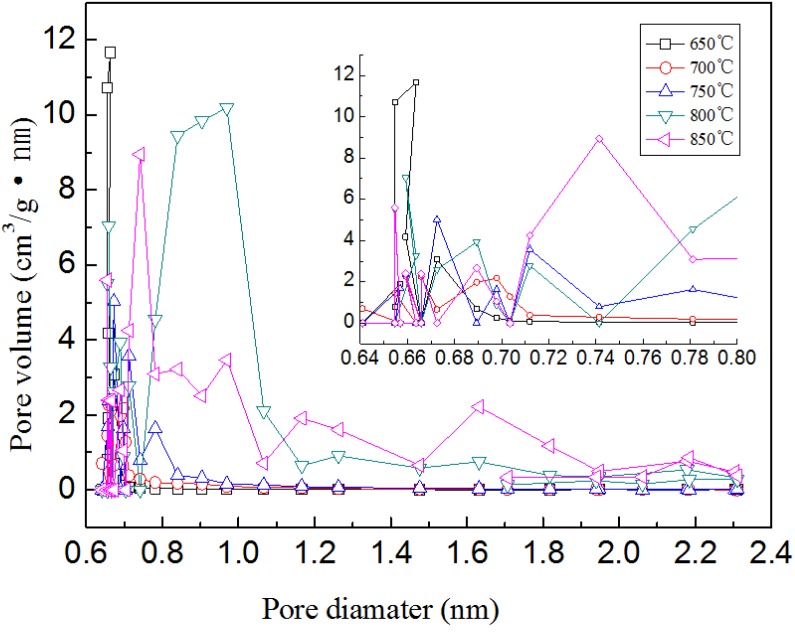
The pore size distributions for BACFs (HK method).

The pore size distributions for the BACFs samples calculated from HK supported the above results. As can be seen from Figure 6, the differences in pore size distribution among them were mainly in the region where pore size was smaller than 0.8 nm. The pore size distribution of BACFs above 800 °C was broader than the former three samples, with a significant increase in the number of micopores with a size 0.8–2.4 nm.

The effects of temperature on the surface areas (BET and micropore) and pore volumes (total, micropore, and mesopore) of BACFs are shown in Table 4. The surface areas and pore volumes of BACFs gradually increased with increasing final carbonization temperature from 650 to 850 °C. When the final activation temperature was raised to 850 °C, the *S*_BET_ and *S*_mic_ were found as 2024 and 1275 m^2^/g, respectively; and the *V*_tot_, *V*_mic_ and *V*_mes_ were found as 0.999, 0.569 and 0.416 cm^3^/g, respectively. The BET values are higher than the bamboo activated carbons reported [35,36]. The micro and mesoporosity percentages at the studied temperatures are also given in Table 2. As can be seen from Table 4, the micro and mesoporosities also change with the increasing activation temperature. The microporosity increased from 70.7% to 72.2% and mesoporosity decreased from 29.3% to 27.8% as temperature increased from 650 to 700 °C. After 700 °C activation temperature, the microporosity was decreased to the value of 57%, which accorded with the iodine and MB adsorption in Figure 2. At all final activation temperatures, mixed (microporous/ mesoporous) activated carbon fibers were obtained. According to the results, BACFs having high-surface areas (S_BET_ and S_mic_) and pore volumes (*V*_tot_, *V*_mic_, and *V*_mes_) was obtained at final activation temperature of 850 °C.

**Table 4:** Surface areas and pore volumes of BACFs at different temperatures.

Temperature (°C)	*S*_BET_ (m^2^/g)	*S*_mic_ (m^2^/g)	*V*_tot_ (cm^3^/g)	*V*_mic_ (cm^3^/g)	*V*_mes_ (cm^3^/g)	*V*_mi_/*V*_t_ (%)
650	483	386	0.256	0.181	0.075	70.7
700	561	449	0.293	0.212	0.081	72.2
750	724	601	0.411	0.291	0.131	70.9
800	1125	856	0.566	0.387	0.165	68.4
850	2024	1275	0.999	0.569	0.416	57.0

Note: *S*_BET_, BET surface area; *S*_mic_, micropore surface area; *V*_tot_, total pore volume; *V*_mic_, micropore volume; *V*_mes_, mesopore volume.

## 3. Experimental

### 3.1. Samples

The mixture of bamboo powder (20 g, Zhangji Corporation, Nanjing, China), phenol (120 g, Jiangtian Corporation, Tianjin, China) and H_3_PO_4_ (9.6 g, Jiangtian Corporation, Tianjin, China) were loaded into a round bottom flask, and heated at 160 °C for 150 min to liquefy bamboo. Subsequently, hexamethylenetetramine (5 wt%) was added to the liquefied medium and heated to 130 °C in 40 min to prepare spinning solution. The spinning solution was spun into filaments by melt-spinning. The spun filaments were cured by soaking in an acid solution (HCHO and HCl, 1:1 by volume, Jiangtian Corporation, Tianjin, China) at 95 °C for 4 h, washed with distilled water and finally dried at 90 °C for 45 min to obtain the fibers. The activation was carried out in a tube furnace and the samples were heated from room temperature to the final activation temperature with a heating rate of 5 °C/min in N_2_. Thereafter, the fibers were held at this temperature for 40 min by introducing a steam flow of 8 g/min and then cooled down to room temperature. These samples were denoted BACFs.

### 3.2. Measurements

The surface morphologies of BACFs were examined using a SEM (SS-550, Shimadzu Corporation, Kyoto, Japan) with an acceleration voltage of 15 kV.

The crystal structures of BACFs were measured using a Powder X-ray Diffractometer (D/max-2500, Rigaku Corporation, Tokyo, Japan) using Cu Kα radiation (λ = 0.154 nm, powdery samples), diffraction angle range of 2θ = 5°−60° with a count time of 20 s at each point. The accelerating voltage and applied current were 40 kV and 100 mA, respectively.

In order to examine the differences of microcrystalline structure of BACFs at various temperatures, the apparent crystallite thickness (*L*_c_), the apparent layer-plane length parallel to the fiber axis (*L*_a_), and the average interlayer spacing *d* were calculated using the Bragg and Scherrer formula. The formulas can be expressed as:

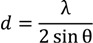
(1)

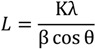
(2)
where θ is the Bragg angle of peaks (°), λ is the wavelength of X-ray used (0.154 nm), and β is peak width at half-height (rad). The form factor K is 0.89 for *L*_c_, and 1.84 for *L*_a_, respectively.

The graphitization degree of BACFs can be calculated by the layer spacing *d*_002_, the simplified formula is:

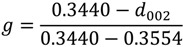
(3)
where: *g*-graphitization degree (%); 0.3440-layer spacing of not graphited carbon material completely (nm); 0.3354-layer spacing of ideal graphite crystal (nm); *d*_002-_layer spacing (nm).

X-ray photoelectron spectroscopy measurements of the samples at various calcination temperatures were carried out on a Kratos Axis UltraDLD (XPS, Shimadzu Corporation, Kyoto, Japan) with a monochromated Al Kα X-ray source (*hv* = 1486.6 eV). XPS survey spectra were recorded with pass energy of 80 eV, and high resolution spectra with pass energy of 40 eV. 

Specific surface area was obtained from a N_2_ adsorption-desorption isotherm taken at 77 K with a Micromeritics ASAP-2020. The surface area of each specimen was obtained based on the Brunauer-Emmett-Teller (BET) method. The micropore area and micropore volume were calculated by t-plot. The micropore size distributions were obtained based on the Horvath-Kawazoe (HK) method. The total pore volume and radius were based on the assumption that nitrogen filled the sample pores at a relative pressure of 0.99 atm.

The iodine and MB adsorption were calculated following GB/T 12496.8-1999 and 12496.10-1999, respectively.

## 4. Conclusions

Bamboo can be effectively used as a raw material for the preparation of high-surface area ACFs after liquefaction and curing. In addition, the raw material is found in abundance and therefore the ACFs cost is expected to be economical. Also, the obtained ACFs with high surface area in this study can be used as a very promising adsorbent for pollution control and other applications.
